# A Scoping Review of Constructs Measured Following Intervention for School Refusal: Are We Measuring Up?

**DOI:** 10.3389/fpsyg.2020.01744

**Published:** 2020-08-20

**Authors:** David Heyne, Johan Strömbeck, Katarina Alanko, Martin Bergström, Robin Ulriksen

**Affiliations:** ^1^Institute of Psychology, Leiden University, Leiden, Netherlands; ^2^Magelungen Utveckling AB, Stockholm, Sweden; ^3^Faculty of Arts, Psychology and Theology, Åbo Akademi University, Turku, Finland; ^4^School of Social Work, Lund University, Lund, Sweden; ^5^BI Norwegian Business School, Oslo, Norway

**Keywords:** school refusal, intervention, outcome, scoping review, guidelines, assessment, children, adolescents

## Abstract

Reviews of the effectiveness of interventions for school refusal (SR) rely upon well-conducted primary studies. Currently there are no guidelines for those conducting primary studies about the measurement of outcome following intervention for SR. Most people would agree that it is important to measure school attendance as an outcome but there has been little discussion about other constructs that warrant measurement. To facilitate this discussion and support the development of guidelines, we conducted a scoping review of constructs measured in studies evaluating intervention for SR. We screened the title and abstract of 3,213 publications found in peer-reviewed journals between 1980 and 2019. After full text review of 271 publications, 50 publications describing 51 studies were included. Results address the frequency with which constructs were measured, along with instruments used, informants, and time-points for measurement. Based on the results, we offer guidelines for choosing constructs to measure following intervention for SR and considerations for how to measure the constructs. Guidelines can increase consistency across primary studies, with benefits for future meta-analyses and international comparisons. They also provide support for practitioners contemplating routine evaluation of their interventions for SR. Ultimately, a core outcome set for SR can be developed.

## Introduction

When a young person^1^ is reluctant or refuses to attend school because of emotional distress, this is referred to as school refusal (SR; Heyne et al., 2019). The emotional distress may take various forms (e.g., excessive fearfulness, depressive affect, temper tantrums, unexplained physical symptoms), and the reluctance or refusal may result in late arrival, occasionally missing whole days, or missing consecutive weeks, months, or years (Heyne et al., 2019). Because SR is often associated with absence from school, it can negatively impact academic achievement (Gottfried, 2014; Gershenson et al., 2017) and socioemotional outcomes (Malcolm et al., 2003; Gottfried, 2014). School absenteeism predicts school drop-out (Schoeneberger, 2012) which is predictive of unemployment (Attwood and Croll, 2006). It is suggested that SR can greatly impact a youth’s quality of life (Torrens Armstrong et al., 2011) and that families are affected when a young person has difficulty going to school (Bryce and Baird, 1986). School absenteeism also places extra burden on school staff (Thornton et al., 2013; Balu and Ehrlich, 2018).

SR occurs among 1–7% of youth in the general population and 5–16% of youth seen in clinical settings (Egger et al., 2003; Heyne and King, 2004; Steinhausen et al., 2008; Havik et al., 2015). SR is a complex problem (Ollendick and King, 1998) associated with a broad range of interacting risk factors (Ingul et al., 2019) and there is a long history of research on SR (Heyne et al., 2019). Interventions have been developed within different disciplines (Heyne, 2006) and evaluated in randomized controlled trials, non-randomized trials, multiple baseline case series, and case studies (see Heyne et al., 2002; Pina et al., 2009; Maynard et al., 2018).

There is a great need to build the knowledge base around interventions for absenteeism (Heyne, 2019) and for SR more specifically (Elliott and Place, 2019). For example, in the field of SR there are questions about the benefits of combining psychosocial and pharmacological interventions (Melvin and Gordon, 2019), the effectiveness of alternative educational programs (Brouwer-Borghuis et al., 2019), ways to improve outcomes for socially anxious youth not helped by current interventions (Heyne et al., 2015), and the long-term effects of intervention (Elliott and Place, 2019). Rigorous evaluation of interventions is needed to answer such questions (Tonge and Silverman, 2019).

Building a meaningful evidence base for SR interventions requires that those who evaluate interventions carefully consider the constructs of interest when measuring outcome. There are lists of assessment instruments and procedures for school attendance problems (Inglés et al., 2015; Kearney, 2016) and SR (Heyne and Rollings, 2002; Ingul et al., 2019) but these lists provide researchers and practitioners with minimal guidance about which constructs are most important when evaluating intervention. Narrative reviews and a systematic review of interventions for SR signal constructs of potential interest, but those reviews are limited in scope. For example, the Maynard et al. (2018) systematic review and meta-analysis of psychosocial interventions for SR reported on post-treatment school attendance and youth anxiety while other outcomes were not evaluated. Pina et al.’s (2009) narrative synthesis of the efficacy of psychosocial interventions for SR covered a broader range of outcomes, including depression and disruptive behavior. However, their review and the narrative reviews of others (Elliott and Place, 2019; Melvin and Gordon, 2019) aimed to synthesize data on the effectiveness of SR interventions and not to identify the range of constructs measured as outcomes.

There has been little discussion about which outcomes to include in the evaluation of intervention for SR, unlike in other fields (e.g., social-emotional learning; Ura et al., 2019). It is thus not surprising that there are no guidelines for the evaluation of intervention for SR comparable to those in other fields (e.g., outcome measures recommended for people with depression and anxiety; Obbarius et al., 2017). Guidelines can enhance the evidence base for SR interventions by ensuring that important constructs are measured, in a consistent way, benefitting comparisons across studies, including future meta-analyses. This, in turn, enhances clinical decision-making. Guidelines also enhance the efficiency with which researchers and practitioners can choose constructs to measure.

The aim of the current study was to support the development of guidelines for measuring outcome following intervention for SR. The primary research question was: Which constructs have been reported in studies evaluating intervention for SR? A secondary question was: How have these constructs been measured? We conducted a review of literature across the last 40 years, undertaking a scoping review rather than a systematic review and meta-analysis. First, scoping reviews are used for reconnaissance (Peters et al., 2015), undertaking a broad review to clarify concepts in a research area, report on the nature of research activity and types of evidence being gathered, and identify gaps (Arksey and O’Malley, 2005; Peters et al., 2015). Second, we did not seek to answer clearly defined questions typically addressed via systematic review and meta-analysis such as the effectiveness of treatment based on the quantitative synthesis of empirical evidence (Pham et al., 2014; Peters et al., 2015). Nor did we examine the methodological quality of included studies, a procedure reserved for systematic reviews (Tricco et al., 2018). Scoping reviews also differ from integrative reviews inasmuch as the latter may combine data from theoretical and empirical literature (Whittemore and Knafl, 2005) which was beyond the scope of the current study.

## Methods

### Inclusion Criteria

Studies were eligible for inclusion if they met the following criteria: (1) Language: published in Danish, Dutch, English, Finnish, Norwegian, or Swedish, the languages in which the authors are fluent; (2) Year: published between 1980 and February 2019 inclusive; (3) Type: published in a peer-reviewed journal, excluding conference abstracts and letters to the editor; (4) Accessibility: for full-text screening, studies needed to be accessible online or in libraries accessible to one of the authors; (5) Design: any study evaluating intervention for SR^2^ (except reviews, study protocols, publications about intervention to prevent the onset of SR, and studies only addressing the prediction of outcome), such as randomized controlled trials, quasi-experimental designs, single case studies, and follow-up studies (even if the follow-up sample was included in an earlier study); and (6) Population: youth in primary or secondary school, between 5 and 18 years, who displayed at least the first three SR criteria presented by Heyne et al. (2019) even if other terms had been used to refer to SR (e.g., school phobia, school refusal behavior). Exclusion of studies occurred according to the order of the criteria presented (e.g., if a study fulfilled criteria 1, 2, 3, and 4, but not criterion 5, it was not screened according to criterion 6).

### Data Sources

A systematic search of 13 databases was conducted between January and February of 2019. Search terms were modified by author MB and a specialist librarian according to the database’s thesaurus or subject terms (see Appendix A). The search yielded 6,437 publications: Academic Search Complete (1,188), Campbell Library (2), CENTRAL (272), Cinahl (672), Cochrane Database of Systematic Reviews (7), DARE (17), ERIC (319), HTA (3), PsycInfo (1,209), PubMed (1,245), Social Science Citation Index (1,238), Social Care Online (9), and SocIndex (256). This search of the 13 databases was supplemented by a search of the reference lists of published systematic reviews of intervention for SR, yielding another 17 publications. In line with the “snowball” technique (Pham et al., 2014) the authors identified 8 additional publications cited in publications already identified via the systematic search process. After removing duplicates, 3,213 publications were available for screening.

### Screening

A three-step screening process was used to establish the relevance of the 3,213 publications. Consensus meetings involving all authors were held at each step to discuss and resolve conflicts.

At Step 1, publication title and abstract were reviewed according to the eligibility criteria specified above. Each publication was independently reviewed by two researchers, with all five authors working in changing pairs. When pairs were unsure whether selection criteria were met, an inclusive approach was employed whereby the publication was included for full-text review at Step 2. This approach was considered appropriate because of the long history of confused terminology in the field of SR, whereby terms other than “school refusal” have been used to describe the phenomenon (see Table 1 in Heyne et al., 2019). For example, publications with titles or abstracts that referred to “anxiety and school attendance/absence” or “somatization and school attendance/absence” were included for screening at Step 2. At the conclusion of Step 1, 271 publications had been selected for full-text review. Rayyan (Ouzzani et al., 2016) is a web-based program to facilitate systematic reviews, and this was used to manage selection at Step 1.

**TABLE 1:** Characteristics of studies evaluating intervention for school refusal.

Characteristic	N (%)	Characteristic	N (%)
**Publication year**		**Sample Size**	
1980–1989	9 (18)	<10	27 (53)
1990–1999	15(29)ab	10–49	15(29)ab
2000–2009	14(27)b	≥50	9(18)b
2010–2019	13 (25)	**Mean Age^d^**	
**Country^c^**		6–9	6(15)b
USA	19(37)ab	10–14	29(73)ab
Australia	10(20)b	15–19	5 (13)
UK	7 (14)	**Gender (% Male)**	
Japan	5 (10)	≤33	10 (20)
Netherlands	3 (6)	34–66	23(45)ab
India	2 (4)	≥67	15 (29)
China	1 (2)	Not specified	3 (6)
Finland	1 (2)	**Intervention**	
Singapore	1 (2)	Psychosocial (other than CBT)	13 (25)
Spain	1 (2)	CBT	12(24)b
Sweden	1 (2)	Behavioral	10 (20)
**Language**		Not specified	4 (8)
English	51 (100)	Medication + other	4 (8)
Danish	0 (0)	CBT + psychosocial^e^	3 (6)
Dutch	0 (0)	Medication + CBT	2(4)b
Finnish	0 (0)	Medication alone	2(4)a
Norwegian	0 (0)	Virtual reality	1 (2)
Swedish	0 (0)		
**Type of study**			
Case study	24 (47)		
Group	19 (37)		
Follow-up only	8 (16)		

*^*a*^Two studies were reported in one publication. ^*b*^The sample was reported in more than one publication. ^*c*^Based on the location of the first author. ^*d*^Follow-up studies were excluded. ^*e*^Chhabra and Puar (2016) employed psychosocial interventions alongside CBT, namely narrative therapy plus counseling with family, teachers, and peers. Last et al. (1998) compared CBT with an educational-support therapy. Tolin et al. (2009) employed CBT and other interventions as needed, such as motivational interviewing.*

At Step 2, full-text review, two researchers independently reviewed each publication, also conducted in changing pairs. Conflicts occurred for 31 of the 271 publications reviewed (11%) and these conflicts were resolved by consensus discussion. During Step 2 it became apparent that some publications used data from a sample reported in an earlier publication. Twenty publications were reviewed a second time and eight were excluded after consensus discussions between the authors (see Appendix B). For example, we excluded a publication that described the longer-term functioning of adults who had refused to attend school during their youth. The authors did not state or imply that the aim of their follow-up was to evaluate *intervention* for SR (Flakierska-Praquin et al., 1997) thus failing to fulfill inclusion criterion 5. It seemed that the aim of their study was to report on longer-term functioning in a naturalistic follow-up.

At Step 3, 33 case-related publications were re-reviewed, this time by two authors working collaboratively (DH, RU). Step 3 was included because it became apparent during Step 2 that publications about case-related material differed considerably in the extent to which outcome was reported. For example, while some publications presented empirical single case studies with a clear focus on outcome (Hagopian and Slifer, 1993) others described real or hypothetical cases simply to illustrate a particular issue such as case conceptualization, while not evaluating the intervention (Hadi et al., 2014). Case-related publications were retained if the title, abstract, or introduction stated or implied that the aim of the study was to evaluate an intervention for SR. Nine of the 33 case-related publications were excluded (see Appendix C). At the conclusion of Step 3, 50 publications were included for data extraction. At all steps, further duplicates were removed once identified.

### Data Extraction

An Excel spreadsheet was developed (JS) to record data extracted from the selected publications. During a consensus meeting with all authors the spreadsheet was reviewed, and minor modifications were made. Data to be included in the spreadsheet were study characteristics (e.g., year of publication, country, type of study, type of intervention evaluated), instruments used to measure outcome, and methodological characteristics (e.g., informants, measurement time-points). All instruments used at post-treatment or follow-up were assumed to be measures of outcome, because studies did not consistently state the purpose of each instrument. Authors JS, KA, and DH independently extracted data for inclusion in the spreadsheet. Consensus meetings were used to resolve uncertainties.

### Data Synthesis

The constructs that were measured following intervention were deduced from the titles of the instruments reported in the studies (authors DH, JS, RU). When unsure, authors conducted searches in Web of Science and Google to clarify the nature of the construct(s) measured via a particular instrument. Descriptive statistics (frequencies, percentages, means, standard deviations) were used to summarize the data.

## Results

**FIGURE 1:**
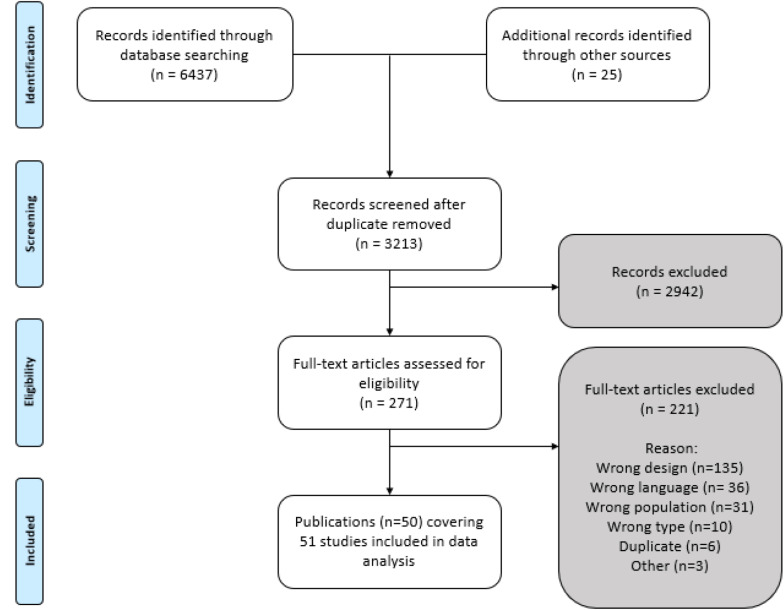
Study selection flowchart modified from Moher et al. (2009).

Fifty publications met the inclusion criteria, one of which comprised two separate studies, yielding a total of 51 studies (see Figure 1).

### Study Characteristics

Table 1 summarizes the characteristics of the 51 studies. Studies were conducted in the USA (37%), Europe (25%), Australia (20%), and Asia (18%), and almost half (47%) were case studies. There were 19 group-based studies (37%), comprising randomized controlled trials (18%), non-randomized controlled trials (4%), and single arm studies with pre-test and post-test but no control group (16%)^3^. Eight studies (16%) only reported follow-up. The mean number of youth per study type was 36 for group-based studies, 48 for follow-up studies, and 2 for case studies. Excluding follow-up studies, the age of the youth ranged between 6 and 18 years (*M* = 12.7; *SD* = 2.4). Across all studies, 55% of youth were males.

Collectively, cognitive behavioral therapy (CBT; 24% of studies) and behavioral intervention (20%) were the most common types of intervention. Other psychosocial interventions were evaluated in 25% of the studies, including multimodal treatment, Morita therapy, parent counseling, collage therapy, and hypnosis. Medication was evaluated as a stand-alone intervention (4% of studies), in conjunction with CBT (4%), or in conjunction with other interventions (8%) such as individual psychotherapy for the child and casework with parents (Berney et al., 1981).

### Constructs Measured as Outcome of Intervention

The constructs measured as outcome are found in Table 2. Among the 29 constructs, school attendance was the most common (73% of studies). Other relatively common constructs were anxiety (39%), depression (37%)^4^, emotional and behavioral symptoms (37%), global functioning (29%), fear (20%) and/or fear of school (16%), and self-efficacy (16%). Constructs measured infrequently included the function of the refusal to attend school (4%), self-esteem (4%), self-concept (2%), psychological well-being and stress (2%), quality of life (2%), and social adjustment (2%). All but two constructs pertained to characteristics of the young person, namely parent self-efficacy for managing school attendance problems, and parent desire for their child to return to school, measured via a single item.

**TABLE 2:** Constructs measured after intervention for school refusal and instruments used to measure the constructs.

	Group *N* = 19	FU *N* = 8	CS *N* = 24	Total *N* = 51
**School attendance**	**16**	**3**	**18**	**37**
**Anxiety**	**12**	**2**	**6**	**20**
Children’s Manifest Anxiety Scale (CMAS/RCMAS)	7	0	5	12
State-Trait Anxiety Inventory (STAI/STAIC)	3	1	2	6
Multidimensional Anxiety Scale for Children (MASC)	2	0	2	4
Anxiety Rating for Children (ARC/ARC-R)	2	1	0	3
Social Anxiety Scale for Children (SASC/SASC-R)	1	0	1	2
Self-Rating Anxiety Scale (SAS)	1	0	0	1
**Depression**	**10**	**2**	**7**	**19**
Children’s Depression Inventory (CDI)	8	0	6	14
Children’s Depression Rating Scale (CDRS/CDRS-R)	2	1	0	3
Beck Depression Inventory (BDI)	1	0	1	2
Self-rating Depression Scale (SDS)	1	0	1	2
Zung Depression scale	0	1	0	1
**Emotional and behavioral symptoms**	**9**	**2**	**8**	**19**
Child Behavior Checklist (CBCL)	7	1	7	15
Teacher’s Report Form (TRF)	3	0	3	6
Youth Self-Report (YSR)	1	0	1	2
Achenbach Young Adult Self-Report (YASR)	0	1	0	1
Devereux Behavior Rating Scales–School Form	0	0	1	1
Rutter Behavior Rating Scales (RBRS)	1	0	0	1
Strengths and Difficulties Questionnaire (SDQ)	1	0	0	1
Young Adult Behavior Checklist (YABCL)	0	1	0	1
**Global functioning**	**10**	**1**	**4**	**15**
Global Assessment of Functioning Scale (GAF)	6	0	1	7
Children’s Global Assessment Scale (CGAS)	4	0	0	4
Clinical Global Impression (CGI)	2	0	2	4
Subjective Units of Distress (SUDS)	1	0	1	2
Comprehensive Psychopathology Rating Scale (CPRS)	0	1	0	1
**Fear**	**7**	**1**	**2**	**10**
Fear Survey Schedule for Children (FSSC-2/FSSC-R)	7	0	2	9
Fear Questionnaire	0	1	0	1
**Fear of going to school/school-related fear**	**5**	**0**	**3**	**8**
School Fear Thermometer (SFT)	4	0	3	7
School-Related Fears Inventory^*a*^ (IME)	1	0	0	1
**Self-efficacy for school-related situations**	**5**	**0**	**3**	**8**
Self-efficacy Questionnaire for School Situations (SEQ-SS)	5	0	3	8
**Diagnosis**	**5**	**1^*b*^**	**2**	**8^*b*^**
Anxiety Disorders Interview Schedule for Children (ADIS-C/P)	3	0	1	4
Diagnostic Interview for Children and Adolescents – Revised (DICA-R)	0	1	0	1
Diagnostic Interview Schedule for Children (NIMH DISC 2.3)	0	1	0	1
Missouri Assessment of Genetics Interview for Children (MAGIC)	1	0	0	1
Schedule for Affective Disorders and Schizophrenia for School-Age Children (K-SADS-P)	1	0	0	1
Unspecified diagnostic interview	0	0	1	1
**Anxiety and depression^*c*^**	**0**	**2**	**1**	**3**
Leeds Anxiety and Depression Scale	0	2	0	2
Revised Child Anxiety and Depression Scale (RCDAS)	0	0	1	1
**Adverse effects of medication**	**1**	**0**	**1**	**2**
New York state psychiatric institute side effect form	1	0	0	1
UKU-scales (side-effects)	0	0	1	1
**Function of refusal to go to school**	**1**	**0**	**1**	**2**
School Refusal Assessment Scale (SRAS)	1	0	1	2
**Outcome of services – general health, social functioning**	**2**	**0**	**0**	**2**
Health of the Nation Outcomes Scales Child and Adolescent (HoNOSCA)	2	0	0	2
**Self-esteem**	**1**	**1**	**0**	**2**
Rosenberg Self Esteem Scale	0	1	0	1
Self-esteem Inventory	1	0	0	1
**Severity of diagnosis**	**0**	**0**	**2**	**2**
Clinical Severity Rating (part of ADIS)	0	0	2	2
**Cognitive and behavioral dimensions in motivation and engagement**	**1**	**0**	**0**	**1**
Motivation and Engagement Scale – High School version (MES-HS)	1	0	0	1
**Consumer satisfaction + parent/adolescent desire for school return**	**1**	**0**	**0**	**1**
School Refusal Program Consumer Satisfaction Questionnaire (SRP-CSQ)	1	0	0	1
**Daily hassles**	**0**	**0**	**1**	**1**
Daily Life Stressors Scale (DLSS)	0	0	1	1
**Dimensions of personality**	**1**	**0**	**0**	**1**
Junior Eysenck Personality Questionnaire (JEPQ)	1	0	0	1
**Mental health**	**0**	**1**	**0**	**1**
General Health Questionnaire (GHQ 30)	0	1	0	1
**Overall improvement since start of intervention**	**1**	**0**	**0**	**1**
Global Improvement Scale	1	0	0	1
**Parent self-efficacy for managing school attendance problems**	**1**	**0**	**0**	**1**
Self-efficacy Questionnaire for Responding to School Attendance Problems (SEQ-RSAP)	1	0	0	1
**Personal functioning**	**1**	**0**	**0**	**1**
Personal Performance Scale (PPS)	1	0	0	1
**Psychological well-being and stress**	**1**	**0**	**0**	**1**
General Well-being Scale	1	0	0	1
**Psychopathology (dimensional)**	**0**	**1**	**0**	**1**
Maudsley Symptom Checklist	0	1	0	1
**Quality of life**	**1**	**0**	**0**	**1**
KIDSCREEN-27	1	0	0	1
**Reading ability**	**1**	**0**	**0**	**1**
Burt Reading Test (BRT)	1	0	0	1
**Self-concept (intrapersonal competence)**	**0**	**0**	**1**	**1**
Piers-Harris Self-concept Scale (P-H)	0	0	1	1
**Social adjustment**	**0**	**1**	**0**	**1**
Social Adjustment Scale	0	1	0	1

*FU, Follow up; CS, Case study. ^*a*^The Spanish title, reported in an English-language publication, is Inventario de Miedos Ecolares (IME).^*b*^One follow-up study used two different diagnostic interviews.^*c*^The constructs “anxiety” and “depression” were measured via a single instrument.*

### Methods Employed to Measure the Constructs

Table 2 also presents the instruments used to measure constructs. It should be noted that school attendance was not measured via a specific instrument *per se*. Moreover, while 37 studies (73%) included school attendance as an outcome measure, information about the process for gathering attendance data was provided in just 16 of the 51 studies (31%). Attendance was reported in a variety of ways, including the number of days or weeks absent, the percentage of time absent, or via qualitative descriptions (e.g., “at 6 months Rob was spending full days in school”; Phillips and Wolpe, 1981).

The other 28 constructs were measured in many different ways. Across the 51 studies we identified 57 instruments used to measure outcome, the most common being the Child Behavior Checklist (Achenbach, 1991a) in 29% of studies, Children’s Depression Inventory (Kovacs, 1992) in 27%, Children’s Manifest Anxiety Scale or its revision (CMAS, RCMAS; Reynolds and Richmond, 1985; Reynolds and Richmond, 2008) in 24%, Fear Survey Schedule for Children (FSSC; Ollendick, 1983) in 18%, Self-Efficacy Questionnaire for School Situations (Heyne et al., 1998) in 16%, School Fear Thermometer (Heyne and Rollings, 2002) in 14%, and Global Assessment of Functioning Scale (GAF; American Psychiatric Association, 1994) in 14%.

Table 3 summarizes the methods, informants, and time-points employed across the 51 studies. Excluding the gathering of attendance data, the most common data gathering methods were questionnaires (59%), clinician rating scales (35%), various types of interviews which were not described as diagnostic interviews (33%), other unspecified rating scales (24%), and interviews described as having a diagnostic purpose (16%). These diagnostic interviews were usually structured [e.g., Anxiety Disorders Interview Schedule for Children (ADIS-C/P); Silverman and Albano, 1996] but in one case an unstructured diagnostic interview was used (Hagopian and Slifer, 1993). Just one study, a case study (Conoley, 1987), employed an observational method to collect data, whereby a teacher observed the child in the classroom. In another case study the young person and parent were instructed to keep a diary (Chorpita et al., 1996).

**TABLE 3:** Methodological characteristics of studies evaluating outcome of intervention for school refusal.

	**Group *N* = 19**	**FU *N* = 8**	**CS *N* = 24**	**Total *N* = 51**
**Measurement method**				
School attendance data	16	3	18	37
Questionnaire	16	5	9	30
Rating scale – clinician	12	2	4	18
Interview – other^a^	7	6	4	17
Rating scale – other^b^	6	0	6	12
Interview – diagnostic	5	1	2	8
Review of medical record	2	3	0	5
Other	3	1	1	4
Diary	0	0	1	1
Observation	0	0	1	1
Test	1	0	0	1
**Informants**				
Youth	17	5	9	31
Parent	11	2	9	22
Clinician	13	2	3	18
School personnel^c^	4	2	3	9
**Time-points**				
Pre-post and follow up	8	0	9	17
Only follow up	1	8	0	9
Daily/weekly	0	0	7	7
Not specified	0	0	7	7
Pre-post	5	0	1	6
Pre + after certain time^d^	5	0	0	5

*^*a*^These interviews were not clearly for diagnostic purposes and were simply described as a structured or unstructured interview, clinical interview, telephone interview, etcetera. ^*b*^Rater by informant other than clinician (e.g., youth). Teachers or school counselors. ^*d*^Measurement at pre-intervention and then another time-point (e.g., after 4 and 8 weeks) which is not post-intervention.*

With respect to informants, some studies used a multi-informant approach by eliciting information about outcome from two informants (20% of studies), three informants (25%), or four informants (6%). Respondents were youth in less than two-thirds of studies (61%), parents in less than one-half of studies (43%), and clinicians in just over one-third of studies (35%). In only nine studies (18%) did teachers or other school personnel report on outcome.

There was considerable variability in the time-points for measuring outcome. Of the 43 group-based studies and case studies (i.e., excluding the 8 studies that were solely follow-up studies), 17 (40%; 8 group-based studies and 9 case studies) involved at least three time-points for gathering data related to outcome: pre-intervention, post-intervention, and follow-up. Eleven of the 43 studies (26%) measured outcome at two time-points (pre-intervention and either post-intervention or another time-point), seven studies (16%; all case studies) included daily or weekly data gathering, and seven studies (16%; all case studies) did not specify the time-points for measuring outcome. The eight follow-up studies, by their very nature, included measurement at some point after intervention ended, ranging from 1 to 20 years. Length of follow-up was 1–5 years in three studies (38% of follow-up studies), 6–15 years in four studies (50%), and 15–20 years in one study (12%).

## Discussion

This is the first review of constructs measured following intervention for SR. Fifty-one studies met inclusion criteria: 9 studies published in the 1980s and 13–15 studies per decade across the last three decades. We discuss the constructs measured in the 51 studies, the way in which they were measured, and the strengths and limitations of the current study. Thereafter we offer guidelines for evaluating outcome following intervention for SR.

### Constructs Measured as Outcomes of Intervention

We identified 29 constructs measured as outcomes. Unsurprisingly, the construct measured most often was school attendance. Other constructs measured with moderate frequency were emotional and behavioral symptoms (including anxiety, fear, school-related fear, and depression), self-efficacy, and global functioning.

#### School Attendance

School attendance is a pivotal measure of outcome following intervention for SR (King et al., 1998) described as a “gold standard” because it provides a real-world referent in ways that psychological rating scales do not (Tonge and Silverman, 2019). This seems to explain why school attendance was the most commonly measured construct.

It is logical that five of the eight follow-up studies did not measure attendance because many participants would have been older than school-age at the time data was gathered. However, one quarter of the case studies did not include school attendance as an outcome even though youth in these studies were aged 6–18 years, so most were probably of school-going age when intervention was completed. Case studies may sometimes be authored by practitioners who find it more difficult to retrieve school attendance data than do research teams that have assistants to contact or visit schools to collect data.

In Maeda’s (2017) case study, attendance data was supplemented with peri-attendance information about the need for parents to escort the young person to school, probably because the therapy evaluated was Morita therapy which involves parents escorting their child to school. Maeda’s study signals the importance of not only determining whether a young person is at school but also how much or little effort is required – by the young person, parents^5^, and school staff – to ensure the young person is at school. Berney et al. (1981) also assessed ability to attend school, taking account of the need for parent escorting, although they noted that information was limited to arrival at school “and does not take into consideration what happens subsequent to that” (p. 112). Mansdorf and Lukens (1987) reported the percent of time two youths displaying SR could remain in school with the parent and alone, during intervention and at 3-month follow-up.

#### Emotional Symptoms

Across the four decades pertinent to this study, definitions of SR have consistently specified the presence of emotional distress (Atkinson et al., 1985; Last et al., 1998; Bernstein et al., 2000; Heyne et al., 2011). It is thus not surprising that, apart from school attendance, the constructs most commonly measured relate to emotional distress (i.e., anxiety, fear, school-related fear, depression, anxiety and depression, and emotional and behavioral symptoms). The two instruments most commonly used to measure anxiety were the CMAS/RCMAS and the State-Trait Anxiety Inventory and its child version (Spielberger et al., 1973). The more recent Multidimensional Anxiety Scale for Children (March et al., 1997) was used in 4 studies and no studies used the Screen for Child Anxiety Related Emotional Disorders (SCARED, Birmaher et al., 1999). Fear was almost always measured with the FSSC or its revision, and fear of school almost always with the SFT. Almost three-quarters of studies measuring depression used the CDI. Emotional and behavioral symptoms were most commonly measured via the CBCL for parent report and the Teacher’s Report Form (Achenbach, 1991b) for teacher report. The Youth Self-Report (Achenbach, 1991c) was only used in two studies, perhaps because of its length.

#### Behavioral Symptoms

Because definitions of SR emphasize emotional distress and specify the absence of severe antisocial behavior, it may seem unsurprising that a little more than one-third of studies measured both emotional *and* behavioral symptoms. However, disorder-level oppositional behavior is reported among 21–44% of youth referred for SR (Heyne et al., 2015) suggesting the importance of measuring oppositional behavior.

#### Self-Efficacy

Youth self-efficacy was measured in 16% of studies, all of which were evaluations of CBT for SR. In each study it was measured as a situation-specific construct via the SEQ-SS, not as a general self-efficacy construct. The SEQ-SS measures a young person’s perception of their ability to cope with school-related situations such as doing school-work, being away from parents, and answering questions about absence. It has been suggested that low self-efficacy for responding to school situations poses a risk for SR and, conversely, high self-efficacy may help explain school attendance even when a young person faces difficult situations at school (Ingul et al., 2019).

One study measured parent self-efficacy using the relatively recent Self-Efficacy Questionnaire for Responding to School Attendance Problems (SEQ-RSAP; Heyne et al., 2007). The study revealed a significant increase in parent self-efficacy following a CBT intervention which included parents (Heyne et al., 2011) providing initial support for measuring the construct of parent self-efficacy when intervention is conducted with parents.

#### Global Functioning, Mental Health, and Diagnosis

Less than one-third of studies measured youths’ global functioning as an outcome, most commonly via the GAF. Mental health is a similarly broad construct, and it was measured in two studies. In four studies the diagnostic status of emotional distress was assessed via the ADIS-C/P, and only four other studies used a diagnostic interview schedule to measure outcome. We observed that a focus on overall adjustment was less typical of case studies than group-based studies. Those conducting group-based studies are typically from research settings where global functioning and diagnosis are commonly assessed.

#### Combinations of Constructs

One study combined outcomes related to attendance and diagnosis to determine the proportion of youth no longer fulfilling operational criteria for SR. Specifically, Heyne et al. (2011) reported that, subsequent to intervention, 55% of youth attended school more than 80% of the time *and* no longer had a diagnosable level of anxiety. This seems to be the first effort to conceptualize outcome as the absence of multiple SR criteria.

#### Constructs Seldom Studied

We could expect that authors measure particular constructs following intervention because those constructs reflect the goals of the intervention. Similarly, constructs measured during intervention and at the end of intervention could be expected to reflect an author’s theory of change. However, during full-text review of studies we noticed that few authors presented a rationale for measuring the constructs embodied in the instruments they used. More often, authors presented a rationale for choosing a specific instrument (e.g., its psychometric properties; suitability for a specific age group). It is thus unclear from this review whether the lack of attention to specific constructs should be interpreted as intentional (e.g., the author believed that the construct was not important enough to be included in a lengthy assessment battery) or unintentional (e.g., the author overlooked the importance of a construct).

We identified numerous constructs that were seldom measured. In some instances, the lack of attention to a construct is understandable. For example, only two studies used the School Refusal Assessment Scale (Kearney and Silverman, 1993) or its revision (Kearney, 2002b) to measure outcome. This instrument was designed to facilitate intervention planning by indicating the function of the refusal to attend school; it was not designed as a measure of outcome. Only two studies measured self-esteem and one study measured self-concept. This might be explained in part by the fact that a large number of studies included in our review evaluated CBT or behavioral intervention. Traditionally, interventions using CBT-based theory and techniques were not focused on raising levels of self-esteem, and if measured, self-esteem was a secondary outcome rather than primary outcome (Kolubinski et al., 2018).

The lack of attention to other constructs is more surprising. Only one study used an instrument focused on social adjustment despite the fact that this construct is linked to SR historically (Buitelaar et al., 1994; Place et al., 2002; Egger et al., 2003) and recently (Ingul and Nordahl, 2013; Blöte et al., 2015; Heyne et al., 2015). We found indirect measurement of social adjustment via global ratings of functioning (e.g., the GAF), a single broad item rated by youth, parents, or clinicians [e.g., “peer relationships” in the HoNOSCA (Gowers et al., 1999)] and questionnaires which simultaneously measured a broad range of constructs (e.g., the CBCL includes a subscale for social problems). However, data from instruments such as the CBCL were usually reported at the broad-band level (i.e., internalizing behavior and externalizing behavior).

No studies measured family functioning as an outcome despite the fact that one-half to two-thirds of families of youth who display SR exhibit maladaptive family functioning, and CBT manuals for SR commonly include family-related work on communication and problem-solving (Heyne et al., 2015). In Bernstein et al. (2001) 1-year naturalistic follow-up study, the Family Adaptability and Cohesion Evaluation Scale II (FACES II; Olson et al., 1982) was used to measure cohesion, adaptability, and family type (balanced to extreme), but only as a predictor of outcome at follow-up.

Only two parent-related constructs were measured as outcomes: self-efficacy in one study, and desire for the child to return to school in another study. Measures of parenting styles and dimensions were not included in any studies, despite the potential impact of parenting on the outcomes of intervention for SR (Heyne et al., 2015). Prabhuswamy et al. (2007) presented pre-intervention data on psychosocial factors such as parental overindulgence and overprotection, but these constructs were not measured post-intervention. Furthermore, no studies reported on parent psychopathology despite the fact that it is often observed in the parents of youth displaying SR (Heyne et al., 2015).

In two recent studies of alternative educational settings for youth displaying SR, authors included positively-oriented constructs. Preece and Howley (2018) noted that staff in an intervention facility for youth unable to attend mainstream school regarded youths’ well-being as essential for re-engagement with formal education, and the General Well-Being Scale (Heubeck and Neill, 2000) was used to monitor youth-reported progress in well-being. McKay-Brown et al. (2019) argued that professionals’ attention to youths’ quality of life, in the context of a wraparound model of care, could enhance SR interventions. Quality of life was measured as an outcome via youth and parent reports on the KIDSCREEN-27 (Kidscreen Group Europe, 2006) which assesses youths’ health and well-being.

### How the Constructs Were Measured

The gathering and reporting of attendance data varied considerably. For example, data was derived via parents’ weekly reports of the number of hours in school (Bernstein et al., 2000) and daily records of attendance kept by teachers (King et al., 1998). Many authors did not specify the source of attendance data so readers cannot assess the reliability of outcomes based on that data. There was also variability in the way attendance data was reported, such as the number of days or weeks youth were absent; the percentage of absence in a period of time (e.g., across 2 weeks; across 4 weeks); achievement of a specific level of school attendance (e.g., at least 80 or 90%); and descriptions such as “at 6 months Rob was spending full days in school” (Phillips and Wolpe, 1981).

Across all constructs, respondents were youth in less than two-thirds of studies, parents in less than one-half of studies, clinicians in just over one-third of studies, and teachers in under one-fifth of studies. Differences emerge when analyzing the data according to study type. For example, while 17 of the 19 group-based studies (89%) elicited youth report, only 9 of the 24 case studies (38%) did so. It is possible that youth had been consulted during outcome evaluation in case studies, but a failure to specify the instrument(s) used to measure outcome led to an underestimation in the current study of the extent to which youth were the informants on outcomes. For example, Hargett and Webster (1996) reported that a young person’s adjustment was monitored over 7 months and that there were no signs of SR, but there was no specification of the data source.

School attendance data aside, the most common methods for data gathering were questionnaires, rating scales completed by clinicians or others, and some form of interview whether for diagnostic or other purposes. We assume questionnaires were regularly used because they are easy to administer, score, and interpret. Even though interviews provide rich qualitative information about the lived experiences of participants, which is important for the development of evidence-based practice (American Psychological Association, 2006), quantitative information grants the most efficient method for comparing results across studies. Just one study used an observational method to gather data following intervention, and just one used a daily diary, despite the recommendation that observations and diaries be used in the assessment of SR (Ollendick and King, 1998).

The time-points for measuring outcome were varied, especially among the group-based studies. The impression we gained during full-text review is that authors rarely if ever justified the time-points they used, so it is difficult to explain the variability. Hargett and Webster (1996) used an ongoing approach to measure outcome, collecting bi-weekly data for 7 months until the end of the school year, and then for the first 2 months of the next school year. This ongoing approach to measurement – albeit easier to conduct in a single case study relative to a group-based study – enabled the authors to demonstrate ongoing efficacy of the intervention, including no relapse back to refusal to attend school or an inability to stay at school after arrival.

### Strengths and Limitations

The scoping review method employed in the current study helped clarify which constructs have been measured as outcomes following SR intervention. We conducted a broad search across four decades, five languages, and various study types (group, case, and follow-up studies). The inclusion of case studies strengthens the relevance of this review for practice-based settings, beyond its relevance for research-based settings.

Unpublished studies were not included, which may have limited the range of constructs identified. At the same time, by restricting our search to peer-reviewed publications we incorporated a crude quality assessment check on the included studies. There was no further assessment of study quality because the aim was to review existing literature according to constructs and methods for measuring constructs, and not to synthesize evidence about intervention effectiveness (Pham et al., 2014).

As is typical of reviews, judgment was used to determine whether studies should be included or excluded. In our review, this included judgment about whether or not case studies met the additional inclusion criterion (i.e., “stated or implied intention to evaluate outcome”), and judgment about which constructs were being measured by the instruments used to evaluate outcome. The latter was necessary because the authors of studies rarely specified which constructs they intended to measure. Judgments were made in pairs, and if there was doubt the research team met for a consensus discussion.

We observed that the SR criteria reported in some studies were unclear. We thus excluded some studies that may well have been evaluations of intervention for SR. To assist future reviews, authors should specify which criteria for SR were (not) applicable to the youth in their study.

It was beyond the scope of this study to review the psychometric properties of the instruments identified across the 51 studies. A review of this kind will benefit decision-making about how best to measure constructs of interest. Existing reviews that contain reliability and validity information about instruments used to measure constructs such as “school engagement” (Fredricks and McColskey, 2012) could be used to guide a review of the psychometric properties of instruments used to measure the constructs included in the guidelines that follow.

### Recommendations

Following, we offer guidelines for measuring outcome following interventions for SR. Greater standardization of outcome measurement – which constructs are measured and how they are measured – facilitates comparison of outcomes across studies and the synthesis of data via meta-analysis. Ultimately, consumers of SR interventions benefit from greater standardization. That is, standardization enhances the accumulation of evidence about the relative benefits of different options for intervention, and practitioners and researchers are more likely to measure and report outcomes that are important to the users of their research (Chu et al., 2015; Kirkham et al., 2016). Guidelines also aid efficiency in decision-making about outcome evaluation. For busy practitioners, this may increase the likelihood that they routinely evaluate progress so as to determine when and how the “scaffolding of support” to youth, families, and schools can be reduced.

#### Guidelines for Choosing Constructs

According to Ollendick and King (1998) professionals often gathered information in certain ways simply because those ways were convenient. We contend that the choice of outcome constructs should be based on the relevance of the constructs to the goals of intervention and not influenced by convenience or habit (e.g., using an instrument because it is familiar).

If a researcher’s or practitioner’s goal is to “simply” help youth return to school (which is seldom a simple process), it might seem logical to limit outcome constructs to school attendance. However, SR is heterogeneous in its etiology and presentation (Heyne and Sauter, 2013; Gallé-Tessoneau and Heyne, 2020) as well as its impact, so intervention should focus on improving broader outcomes for youth, necessitating a wider palette of outcome constructs. In other words, the goals of intervention are likely to include general goals (e.g., increased school attendance) and specific goals informed by case formulation (e.g., increased social involvement).

Assuming researchers and practitioners choose to measure multiple outcomes, which should they be? In a review of SR intervention, Elliott and Place (2019) noted: “Researchers, therefore, need to be explicit about whether the primary outcome sought in their intervention studies is reduction in anxiety or increased school attendance.” Based on the findings in our review and our own reflections on the goals of intervention, we propose that evaluation of outcome includes – but also extends beyond – the constructs of school attendance and anxiety. If we assume that the constructs measured in the 51 studies reviewed here were chosen because they reflected the goals of the interventions offered and not because of convenience or habit, then researchers and practitioners are well advised to measure the more common constructs identified in the current study: school attendance; emotional functioning including anxiety, fear/fear of school, and depression; behavioral symptoms; global functioning; and self-efficacy.

School attendance is an important foundational competency for youth (Kearney et al., 2019a) and a gold-standard, real-world referent for evaluating interventions for school attendance problems (Tonge and Silverman, 2019). It is self-evident that it would be included in evaluations of SR. As noted in section “School Attendance,” peri-attendance variables are also relevant. A smartphone application could be developed to facilitate ecological momentary assessment of variables beyond school attendance and absence, such as the young person’s whereabouts (e.g., in class or often in the school nurse’s office), how much time parents spend at school with a separation anxious youth participating in intervention for SR, and the youth’s emotional distress during the school day.

Emotional functioning includes the youth’s levels of fear, anxiety, and depression. Alongside general levels of emotional distress, researchers and practitioners may measure distress experienced within the school setting. Three relatively recent instruments not yet incorporated in outcome studies have face validity for SR intervention: the School Anxiety Scale–Teacher Report (Lyneham et al., 2008), the School Anxiety Inventory^6^ (SAI; García-Fernández et al., 2011) and its short version (García-Fernández et al., 2014), and the SChool REfusal EvaluatioN Scale (SCREEN; Gallé-Tessonneau and Gana, 2019). These provide more detailed information about youths’ emotional distress in the school context relative to instruments such as the MASC and its revision (MASC-2; March, 2013), the Revised Children’s Manifest Anxiety Scale (Reynolds and Richmond, 1985) and its revision (RCMAS-2; Reynolds and Richmond, 2008), and the Spence Children’s Anxiety Scale (Spence, 1998). The SCARED has a 4-item school phobia subscale but the subscale does not always emerge in analyses of the instrument’s factor structure (Inglés et al., 2015). Because psychosomatic symptoms are also prominent in cases of SR (Heyne et al., 2015) they should be measured alongside the other constructs associated with emotional distress.

Measures of behavioral symptoms can provide an indication of the frequency and severity of a young person’s resistance to school attendance. Parents can be asked to complete a daily logbook that includes ratings of noncompliance and disruption (Kearney and Albano, 2000) and more specifically the child’s resistance to efforts to get them to go to school (Kurita, 1991). Anecdotal evidence suggests that the decrease in a young person’s resistance to school attendance is a very important outcome for parents who are often emotionally and physically exhausted due to resistive behaviors often directed at them.

Global functioning provides a measure of overall outcome following SR intervention. It was infrequently incorporated in case studies included in this review. To benchmark global functioning of youth included in case studies against those included in group-based studies, authors preparing case studies will need to incorporate a measure of global functioning. The most common measure of global functioning was the clinician-rated GAF, which includes assessment of the impact of symptoms on daily life. It is important to also obtain youth, parent, and teacher perspectives on global functioning, perhaps via the impact supplement of the Strengths and Difficulties Questionnaire (Goodman, 1999).

Youth self-efficacy and parent self-efficacy are cognitive constructs relevant to outcome because they are a key target for change during CBT for SR and they have been found to increase following intervention (Heyne et al., 2015). Non-CBT interventions may also have a positive effect on school attendance due to youths’ increased self-efficacy for attending school and/or parents’ increased self-efficacy for responding to a child’s refusal to attend. As such, it is valuable to include youth and parent measures of self-efficacy in the evaluation of CBT and non-CBT interventions. Furthermore, higher levels of self-efficacy at post-intervention may help prevent relapse during a follow-up period because youth and/or parents are more likely to engage in adaptive behaviors during those times when there are small setbacks.

Other constructs found to be less common or not measured at all also warrant attention. Following, we draw attention to constructs relevant to the young person’s adjustment; motivation for change; family functioning; and side effects of intervention. We conclude this section with attention to individualized goals as constructs of interest, and variation in the constructs of interest according to the length of follow-up conducted.

An important facet of the young person’s adjustment is their social adjustment (see section “Constructs Seldom Studied”). An instrument measuring this construct should be used, or at the very least authors should report outcome according to sub-scales that measure social functioning (e.g., “social problems” in the CBCL). We also encourage researchers and practitioners to measure well-being as a broader youth-focused construct, reflecting a holistic perspective on the success of intervention. School engagement is an important construct because of the potential for relapse if there is little school engagement following return to school. McKay-Brown et al. (2019) used the Motivation and Engagement Scale (Martin, 2014) to measure “educational functioning,” noting that the instrument measures adaptive and maladaptive factors related to learning behaviors “that are linked to school engagement” (p. 96). According to Amai (2020) a related construct of “sense of school adaptation” has been widely measured in Japan using Furuichi and Tamaki’s (1994) School Adaptation Scale (e.g., I look forward to going to school; I want to go to school even if I feel a little bad). Other youth-focused constructs that appear to be associated with SR but have not been measured as outcomes include emotion regulation (Hughes et al., 2010) and negative automatic thoughts and thinking styles (Maric et al., 2012). Academic functioning is an important construct when evaluating interventions for absenteeism (Tonge and Silverman, 2019), including SR.

Parent and youth motivation for achieving school return was measured in just one study via a single item about desire for return to school (Melvin et al., 2017). Readiness for change is found to be related to outcome in studies of psychotherapy with adults and adolescents (Krebs et al., 2018) and seems important for understanding treatment progress among depressed adolescents (Rodriguez-Quintana and Lewis, 2019). It should receive more attention in studies of intervention for SR, with measurements at pre-intervention, mid-intervention, and post-intervention, as recommended by Rodriguez-Quintana and Lewis (2019) in relation to adolescent depression.

A systemic perspective on SR and interventions for SR calls for measurement of parenting and family functioning (see section “Constructs Seldom Studied”). Measuring these constructs during intervention, at post-intervention, and at follow-ups will allow us to establish the extent of change in functioning as well as the extent to which change in parent and family functioning is associated with change in school attendance and other outcomes for youth. As noted, no studies included in the current review addressed these constructs. If the word-limit restrictions of journals lead authors to exclude data about parent and family functioning, such information should be included in supplementary online materials or adjunct publications so that the evidence base for changes in these constructs grows.

A few studies of pharmacological intervention measured adverse effects, and some studies of psychosocial intervention measured the experience of intervention. In Head and Jamieson’s (2006) study, which was excluded from our review^7^, the focus of enquiry was broadened from “technical success” in terms of school attendance to whether and why youth, parents, and teachers regarded intervention as successful. Maeda (2012) provided a qualitative account of the impact of intervention (forced school attendance) on the parents: “Thus, intensive exposure therapy for school return could be a burden to children, parents, and school officials in spite of being effective for school return” (p. 309). Information about the experience of intervention may impact consumer uptake and persistence with intervention. Just one study included in our review reported on consumer satisfaction as an outcome. Consumer satisfaction warrants inclusion in all reports on outcome because it provides information that can help shape interventions in ways that enhance uptake, persistence, and outcome.

Individualized goals for intervention constitute important constructs to be measured. In Meyer et al. (1999) case study, “progress toward goals” was measured according to youth and parent behaviors. This approach to conceptualizing change may have been used because the young person had an intellectual disability. Nonetheless, it presents a model for all practitioners evaluating interventions, and where possible, for the evaluation of outcome in group-based studies. Another example of attention to specific goals is found in the work of Kearney and Silverman (1990). They used a standard set of outcome measures across seven cases but they expected differences on the measures per case, depending on the function served by each youth’s refusal to attend school. It was suggested that “perhaps a more appropriate way to examine the data is to focus primarily on those measures that are pertinent to each functional category” (p. 354). Indeed, goal-based outcomes derived from youth and parent goals for intervention should be considered for inclusion in a battery of outcome measures (Law and Jacob, 2015).

The constructs of interest will also vary according to the length of follow-up being conducted. For example, in Flakierska et al. (1988) 15- to 20-year follow-up study of adults who had refused to attend school as youth, constructs included the number of visits to adult outpatient psychiatric care and the number of children they had. Clearly, longer follow-ups call for broader conceptualization of the constructs of interest.

Whichever constructs are measured, authors need to provide a clear rationale for choosing those constructs. This is in contrast to what we observed during the current review. For example, interviews were used in many studies, presumably to measure diagnosis or global functioning, but a clear explanation for why an interview was used was not always included. Our observation that authors often neglected to specify the constructs of interest reflects an unfortunate long-standing phenomenon. Even in the 1980s Valles and Oddy (1984) noted that “measures of outcome are ill-defined” (p. 36). When the specification of constructs of interest becomes standard practice, authors are prompted to reflect on the goals they have for their intervention. Specification of constructs will also benefit future reviews of the effectiveness of interventions for SR. First, it will expedite the selection of primary studies for systematic review because the outcomes of interest are clearly specified in the primary studies. Second, it can facilitate the interpretation of outcomes from meta-analyses. For example, effect sizes might differ between primary studies because of differences in the conceptualization and operationalization of outcome, whereby some measured constructs may be more sensitive to change than others.

#### Guidelines for Measuring Constructs

To advance the evidence base for SR interventions, careful consideration needs to be given to the methods for measuring chosen constructs. First, consistency in the choice of instrument or procedure to measure a construct such as school attendance will facilitate the comparison of outcomes across schools, school districts, and states (Hobbs et al., 2018), as well as across countries. For example, Fredricks and McColskey (2012) noted large variation in how constructs were measured in the field of school engagement, making it difficult to compare findings across studies. Second, when other methodological issues are consistent across studies, such as choice of informants and measurement time-points, then the interpretation of comparative results will be simplified. For example, Melvin and Gordon (2019) conducted a review of antidepressant medication for SR and suggested that study differences in the source of information (e.g., school versus parent) and the timing of assessment created measurement error variance which could explain the apparent lack of benefit when combining medication with CBT for SR. Third, greater consistency in how outcome data is reported will enhance data synthesis such as meta-analysis. In a meta-analysis of the relationship between anxiety and school attendance problems, Finning et al. (2019) concluded that methodological differences across studies limited scope for combining studies. Their study was not a meta-analysis of outcome, but the challenges they experienced in synthesizing data across studies would apply equally to meta-analyses of outcome following intervention for SR.

Following, we discuss six topics relevant to promoting consistency in the evaluation of outcome: accessing valid data on school attendance; using psychometrically sound instruments to measure other constructs; establishing uniformity in the choice of psychometrically sound instruments; establishing uniformity in time-points for measurement; specifying criteria for determining when desired outcome has been achieved; and incorporating various sources of data.

First, those who evaluate outcome need access to school attendance data that accurately describes a young person’s attendance and non-attendance. Because schools are increasingly held to account for the registration of attendance and non-attendance (Hutt and Gottfried, 2019; OFSTED, 2019) it is reasonable to expect that researchers and practitioners could acquire school-based data per half-day, and ideally per lesson. This can be converted to a percentage of school attendance for a specified time-frame [e.g., number of lessons (or hours) attended in a 4 week period divided by the total number of lessons (or hours) scheduled in that time-frame]. When possible, information should also be gathered about peri-attendance variables such as late arrival to school and absence from a lesson whilst still at school (e.g., spending time in the school counselor’s office). Some schools may not (yet) collect such detailed data. To fill this gap, parents and youth can be asked to record attendance as well as peri-attendance variables. Presumably youth can provide a more accurate account of absence from class during attendance at school. To increase parent and youth compliance with the request for data, researchers and practitioners might use automated reminders (e.g., smartphone applications) or diaries managed via email contact. At the end of each week during the post-intervention and follow-up time-frames, youth and parents could be asked to list how many classes were held and which classes were attended. If possible, this would occur at the end of each day to reduce problems with recall at the end of the week. Because there are discrepancies in school- and parent-reported absences (Lomholt et al., 2020) and school- and youth-reported absences (Keppens et al., 2019) we recommend gathering data from all three sources and reporting outcomes for each group separately. Similarities and differences in outcomes based on youth-, parent-, and school-reported absences need to be taken into account when authors discuss the effectiveness of the intervention.

Second, constructs need to be measured via instruments with strong psychometric properties. Simply put, does the instrument measure the construct it is supposed to measure and in a reliable way? Important psychometric properties include construct validity, internal consistency reliability, test-retest reliability, and sensitivity to treatment effects (Spence, 2018). Psychometrically strong instruments are needed for group-wise significance tests when evaluating intervention effects in group-based studies. For case studies, two methods which practitioners can use to analyze clinically meaningful change are “crossing clinical thresholds” and “reliable change” (Wolpert et al., 2015). Such analyses also rely on measurement via psychometrically sound instruments. Other characteristics that render instruments more suitable for measuring outcome following intervention include applicability to a wide age range (e.g., to compare intervention effects for younger versus older students); availability for different respondents (e.g., youth, parents, teachers); availability of normative data and clinical cut-off scores (e.g., to determine whether change is clinically meaningful); and the time taken to administer the instrument. A more fundamental issue is the need to specify the instruments used to gather outcome data; this was not always the case in the studies we reviewed.

Third, when a majority of researchers and practitioners use the same instrument to measure a specific construct, the comparison of outcomes across studies will be more robust. It will also be easier to combine data for meta-analyses, avoiding the methodological complications that occur when different measures of the same construct are used to create a common outcome measure (Bennett et al., 2013). As the field achieves greater consensus on which instruments are most valuable, efforts should be made to ensure instruments are available in many languages via thorough translation and adaptation processes that include forward and backward translation (Van Widenfelt et al., 2005).

Fourth, there should be more uniformity in the time-points for measuring constructs. To illustrate, one study assessed school attendance across the 4 weeks following intervention (Melvin et al., 2017) while earlier studies did so across 2 weeks (Heyne et al., 2011). We contend that the 4-week period provides a better test of the reliability of intervention effects with respect to school attendance, even though a 2-week time-frame is commonly employed in criteria for deciding whether a school attendance problem exists (Kearney, 2008). It is logical to apply the 4-week time-frame to the measurement of all chosen constructs. For example, 4 weeks after delivery of the final component of intervention, researchers or practitioners administer instruments to measure constructs such as anxiety and depression. If the CDI is used to measure depression, then 4 weeks after intervention finished the young person is invited to report symptoms of depression across the prior 2 weeks (the standard time-frame prescribed for administration of the CDI). Longer-term outcome is ideally based on follow-ups at 6 and 12 months after intervention finishes. This permits more robust evaluation of the young person’s adjustment to the ongoing academic and social-emotional challenges of life at school. When interventions are prolonged (e.g., placement within an alternative educational setting), 6- and 12-months evaluations can take place during the course of the intervention, instead of waiting until a 12-month program is complete before evaluating its effects. Finally, alongside standard time-frames for measurement, researchers and practitioners may choose to conduct additional measurement at other time-points.

Fifth, there needs to be consistency in criteria for determining whether desired outcome is achieved, and clear specification by authors of the criteria they used. This will address Kearney and Silverman’s (1990) observation 30 years ago that many studies of intervention for absenteeism used inconsistent or inadequate criteria for positive outcome. The constructs used to measure desired outcome will depend on the stated aims of the intervention. We found that many case studies included qualitative descriptions of outcome (e.g., “attendance and anxiety remained at acceptable levels”; Kearney, 2002a) whereas the group-based studies analyzed outcomes quantitatively (e.g., change in mean level of anxiety), although we also found qualitative descriptions in some group-based studies (e.g., “in general, youth no longer exhibited upset on arrival at school”; King et al., 2001). It is incumbent upon authors to report the proportion of cases fulfilling a specified outcome rather than relying on non-specific terms such as “in general” and “typically.” With respect to school attendance, authors can report on the proportion of youth reaching a specific level of attendance, alongside their reports of the average amount of attendance in the 4-week period since intervention finished. Chronic or persistent absenteeism is increasingly specified as 10% absence or more in a given time-frame (Heyne et al., in press) so a standard criterion for desired outcome would be attendance above 90%. To be able to compare outcomes across case studies and group-based studies, case studies could include a minimum level of quantitative data (e.g., whether or not the young person achieved more than 90% attendance at post-intervention). Authors can also consider combining constructs to determine the proportion of youth who simultaneously fulfill two or more criteria for desired outcome (see section “Combinations of Constructs”).

Sixth, all stakeholders in interventions for SR – youth, parents, education professionals, and helping professionals – should be invited to report on the outcome of intervention to ensure a breadth of perspectives on outcome. In a meta-analysis of five decades of research on psychological interventions for youth, Weisz et al. (2017) argued that “it matters a lot” who reports on outcome, based on the observation that effect sizes differed across informants (p. 94). They emphasized the need for researchers and practitioners to obtain and integrate information from multiple informants and to be explicit about the source of outcome data. This emphasis on multi-source and multi-method assessment is not new to the field of school attendance and absenteeism (Ollendick and King, 1998; Kearney, 2002a). All stakeholders should also be consulted about the constructs that ought to be measured as outcomes following intervention for SR.

## Conclusion

Are we measuring up? In other words, are we as researchers and practitioners measuring outcome in a way that helps to build a meaningful evidence base for SR intervention?

With respect to constructs measured, there has been some consistency across studies but also considerable variability. School attendance is the only construct that was measured in more than two-thirds of the studies. The fact that other “common” constructs (i.e., emotional and behavioral symptoms, anxiety, fear and/or fear of school, depression, self-efficacy, and global functioning) were measured in a third of studies or less might reflect variability in what was regarded as important to measure, but it might also reflect a failure to consider the benefit of measuring such constructs. It is unlikely that the low rate at which these “common” constructs were measured was due to the unavailability of instruments because there have been instruments to measure the majority of these constructs since the 1980s. Unfortunately, authors infrequently provided justification for the choice of constructs measured. With respect to the way in which constructs were measured, there was also substantial variability. It is important that authors of future studies clearly specify the rationale for focusing on specific constructs and for using specific instruments to measure those constructs.

Despite current shortcomings in the evaluation of outcome following SR intervention, the current review yields initial guidelines for researchers and practitioners planning to evaluate outcome. Measurement of the more common constructs identified in this review (i.e., attendance; emotional, behavioral, and global functioning; self-efficacy) can be supplemented with measurement of the young person’s social adjustment and the well-being of the young person, parents, and family. These guidelines may yield greater uniformity in the evaluation of interventions, benefitting science and practice, and thus the youth, parents, and schools impacted by SR.

The current review can also serve as a platform for further work on the development of a core outcome set for SR, possibly via the international consensus-based process presented in COS-STAP (Core Outcome Set-STAndardised Protocol Items; Kirkham et al., 2019). The fact that SR is not included as a disorder in classification systems such as the DSM does not negate the need for a core outcome set. Work on a core outcome set should foster broad stakeholder input, broader than the perspective of the authors of this paper. Attention should be paid to changes in education and technology, such as competency-based education and virtual learning (Kearney et al., 2019b) necessitating a reconceptualization of traditional outcomes such as percentage of time spent at school.

Attention should also be paid to the development of a core outcome set for interventions focused on truancy, the school attendance problem characterized by “skipping” or absconding from school (Heyne et al., 2019). In a systematic review and meta-analysis of the effects of interventions for truancy, Maynard et al. (2013) found considerable variability in how study authors operationalized and reported outcomes related to school attendance, and there was sometimes a lack of clarity about what had been measured (e.g., excused and/or unexcused absences). Maynard and colleagues called for greater consistency in measuring and reporting school attendance when evaluating truancy interventions. Broadening the perspective, Keppens and Spruyt (2020) concluded an integrative review of interventions to prevent truancy with a call to evaluate outcomes other than truancy-related absence, such as graduation in the longer-term. Their review also signals the need to think more broadly about truancy, not simply as a behavior to be changed but also as a symptom of the need for change in school bonding.

In conclusion, measuring up to the task of advancing the science and practice of SR intervention requires greater consensus on the evaluation of outcome. This review contributes to the discussion about guidelines for evaluating outcome. Before a core outcome set becomes available we encourage researchers and practitioners to carefully consider and justify their choice of constructs and measurement methods. A collective effort is also needed to increase consistency in the choice of psychometrically sound instruments for measuring important constructs.

## Author Contributions

All authors were involved in designing the study, conducting the review, and preparing the manuscript.

## Conflict of Interest

The authors declare that the research was conducted in the absence of any commercial or financial relationships that could be construed as a potential conflict of interest.

## Footnotes

1 We use the terms young people and youth to refer collectively to children and adolescents.

2 No attempt was made to select or classify SR interventions according to the tiered system introduced by Kearney and Graczyk (2014) whereby emerging SR (Tier 2) is differentiated from severe or chronic SR (Tier 3) because most studies to date have provided little or no information about the severity or chronicity of SR among youth in the study samples.

3 This totals to 38% due to rounding.

4 Three other studies (6%) measured both anxiety and depression with a single instrument.

5 We use the term “parents” to refer to parents and/or other caregivers.

6 This is an updated version of the Inventario de Miedos Ecolares (IME) presented in Table 2. The IME only assesses anxiety-generating situations whereas the SAI also assesses the three anxiety response systems (cognitive, physiological, and motor or behavioral).

7 The study did not meet inclusion criterion 5; it was not an evaluation of intervention but a report on the experience of intervention.

## Appendix A | Search Terms

“school refus^∗^” (school refusal/school refusing/school refuser) OR “school phobi^∗^” (school phobia/school phobic) OR “anxiety-based school refus^∗^” OR “anxiety-based absen^∗^” (anxiety-based absence/anxiety-based absentee/anxiety-based absenteeism) OR “anx^∗^ school refus^∗^” (anxious school refus^∗^/anxious school refusing) OR “school reluctan^∗^” (school reluctance/school reluctant; as long as the youth are not identified as truanting or showing some other attendance problem) OR “school absen^∗^ (school absence/school absentee/school absenteeism; as long as the youth are not identified as truanting or showing some other attendance problem) OR “school avoidan^∗^” (school avoider/school avoidance/school avoidant; as long as the youth are not identified as truanting or showing some other attendance problem) OR “emotional^∗^ absen^∗^” (emotional absen^∗^/emotionally absen^∗^) OR “school refus^∗^ behav^∗^” (school refus^∗^ behaviour/school refus^∗^ behaviour) OR “separation anx^∗^” (separation anxiety/separation anxious; as long as this was conceptually linked to absence from school or difficulty going to school) AND [Child^∗^ (child/childhood/children) OR adolescen^∗^ (adolescence/adolescent) OR student^∗^ (student/students) OR youth^∗^ (youth/youths) OR young^∗^ (young/youngster) OR teenage^∗^ (teenage/teenager) OR “lower school” OR “upper school” OR “grammar school” OR “high school”] AND [RCT OR experiment^∗^ OR “quasi experiment^∗^” OR “longitudinal^∗^” or “control group^∗^” OR “case control” OR “quasi-experiment^∗^ OR “case stud^∗^” (case study/case studies) OR “follow-up” OR interven^∗^ (intervene/intervening/intervention) OR program^∗^ (program/programme) OR outcome^∗^ OR eval^∗^ (evaluate/evaluated/evaluation)].
